# Volumetric analysis of external root resorption after clear aligner treatment of open bite cases: a CBCT study

**DOI:** 10.1186/s12903-026-09203-8

**Published:** 2026-07-15

**Authors:** Dina Elfouly, Esraa A. Elnadoury, Tarek El-Bialy

**Affiliations:** 1 Department of Orthodontics, Faculty of Dentistry, Alexandria University, Champolion street, Azarita, Alexandria, Egypt; 2 Department of Oral radiology, Faculty of Dentistry, Alexandria University, Champolion street, Azarita, Alexandria, Egypt; 3 Department of Orthodontics, School of Dentistry, Faculty of Medicine and Dentistry, University of Alberta, Edmonton, Canada

**Keywords:** Volumetric, External root resorption, Clear Aligner, Open bite, CBCT

## Abstract

**Background:**

Orthodontically induced external root resorption (OIERR) is a recognized adverse effect of orthodontic treatment. Limited literature exists that reported on volumetric root changes in anterior open bite patients treated with clear aligners. This study aimed to evaluate three-dimensional volumetric changes in anterior teeth roots using cone-beam computed tomography (CBCT) in open-bite patients treated with clear aligners.

**Methods:**

Fifty CBCT scans [pre-treatment (T1) and post-treatment (T2)] obtained from 25 adult patients (11 males,14 females; mean age: 32.67 ± 10.67 years) with anterior open bite (1–4 mm) treated using clear aligners were retrospectively analyzed. Semi-automated segmentation of the twelve anterior teeth was performed using Blue Sky Plan software, followed by three-dimensional superimposition in 3-matic software. A reference line at the cementoenamel junction was used to trim the crown, and root volumes were measured at both time points. Percentage changes in root volume between pre-treatment and post-treatment were calculated. The level of significance was set at *p* ≤ 0.05. Skeletal and dental parameters were assessed before and after treatment. The reproducibility of the measurements was assessed using the intraclass correlation coefficient (ICC).

**Results:**

Both intra-examiner and inter-examiner reliability were excellent (ICC: 0.90–0.94 and 0.92–0.96, respectively; *p* < 0.001). All anterior teeth demonstrated significant reduction in root volume. Maxillary canines showed the greatest percentage decrease (− 6.33 ± 2.58%), followed by the central (− 5.91 ± 4.93%) and lateral incisors (− 5.50 ± 3.24%). Mandibular canines again showed the largest reduction (− 6.75 ± 2.33%), followed by lateral (− 5.58 ± 3.20%) and central incisors (− 4.86 ± 2.54%). Males exhibited significantly greater root volume reduction across all anterior teeth (*p* < 0.05), while no significant correlation was found with age.

**Conclusion:**

Patients with anterior open bite who were treated with clear aligners showed mild root volume reduction in all anterior teeth with canines being most affected. This reduction is generally considered to be clinically insignificant. Male patients showed greater volumetric reduction, whereas age and magnitude of incisor movement were not significantly correlated with root volume changes.

## Introduction

 Orthodontically induced external root resorption (OIERR) is a prevalent iatrogenic effect of orthodontic treatment [1]. Although minor resorption is usually clinically insignificant [2], severe OIERR (> 6 mm in linear measurement) occurs in approximately1.5% of the patients [3] and may compromise long-term tooth prognosis. Numerous factors affect OIERR, including duration of treatment, type and magnitude of force [4], and the amount of tooth movement [2]. Maxillary and mandibular incisors are particularly susceptible to resorption during orthodontic treatment [5]. 

Since its introduction, clear aligner treatment (CAT) has been sought due to its superior aesthetics and patient comfort [6, 7]. Initially indicated for mild to moderate malocclusions, CAT is now increasingly used in complex cases, including anterior open bite treatment [8]. Costa et al. [9] have demonstrated the success of CAT in treating open bite cases, producing a bite-block effect and controlling the vertical dimension. Li et al. [10] and Zhang et al. [11] demonstrated a lower incidence of apical root resorption in patients treated with aligner therapy compared to those treated with fixed appliances. These findings are further supported by a recent systematic review and meta-analysis by Darvizeh et al. [12]

In CAT, tooth movement is achieved through staged activation and aligner material elasticity, often combined with attachments and auxiliary mechanics such as intermaxillary elastics. These factors may influence the magnitude, direction, and distribution of forces applied to the teeth, potentially generating intermittent compressive stress within the periodontal ligament, which may contribute to orthodontically induced root resorption [13]. 

OIERR has traditionally been assessed using linear measurements on two-dimensional radiographs; however, these approaches may underestimate the true extent of structural changes. Cone-beam computed tomography (CBCT) has been shown to be a reliable method for assessing OIERR [14, 15] as it enables accurate three-dimensional volumetric evaluation of root morphology and precise assessment of OIERR than two-dimensional radiographs [16, 17]. 

While grading systems such as the Levander and Malmgren classification [18] have been widely used for linear assessment of root resorption, they are not directly applicable to three-dimensional imaging [19]. Currently, no standardized classification exists for volumetric evaluation. Based on the available literature, volumetric root reductions of less than approximately 10% are generally considered mild and unlikely to significantly affect tooth prognosis, although these thresholds should be interpreted with caution [20]. 

While OIERR associated with CAT has been previously investigated [21, 22], to date, no study has compared the volumetric changes in root morphology among anterior teeth during treatment of open-bite patients using CAT. Therefore, this study aimed to assess and compare volumetric changes in anterior tooth roots using CBCT in open-bite patients treated with CAT. The null hypothesis of this study was that there is no significant difference in the volumetric alterations of the roots of anterior teeth in open-bite patients following treatment with CAT.

## Materials and methods

### Study design and ethical approval

This retrospective study was conducted at the Department of Orthodontics, Faculty of Dentistry, Alexandria University, Egypt, in conjunction with the University of Alberta, Canada. Ethical approval for conducting the study was obtained from the Institutional Review Board of the Faculty of Dentistry, Alexandria University (IORG:0008839, Protocol number 1065-04/2025), and the University of Alberta Health Ethics Review Board protocol number Pro00040543. This study was conducted in accordance with the Strengthening the Reporting of Observational Studies in Epidemiology (STROBE) guidelines for retrospective observational studies [23]. 

### Sample selection

The de-identified records of patients treated by the same health professional (T.E.) were screened for eligibility. The patients had previously granted informed consent for the use of their records for research purposes. The inclusion criteria were: (1) Adult patients (> 18 years), (2) Cases of anterior open bite (edge-to-edge bite or negative overbite), (3) Treatment using a standardized protocol with Invisalign^®^ clear aligners (Align Technology, San Jose, CA, USA), including intrusion of the upper and lower posterior teeth facilitated by vertical elastics applied to upper and lower canines, extrusion of upper anterior teeth using attachments to achieve a final outcome of 50% vertical overlap of the lower incisors by the upper incisors, and the use of Class II/III elastics and interproximal reduction (IPR) in the lower anterior segment when indicated. (4) Hight-quality pre- and post-treatment CBCT scans captured using the same device. (5) Compliant Patients who wore each aligner for 7 days, at least 22 h per day, as recommended by Align technology with regular 6-weeks checkup for monitoring progress. Exclusion criteria included individuals, with a history of trauma, prior orthodontic treatment, incomplete records, or cases treated with tooth extraction, temporary anchorage devices (TADS), surgery, or adjunctive appliances. Patients with systemic diseases, craniofacial anomalies, TMJ disorders, poor oral health, root-filled teeth, open apices, or pre-existing root resorption were also excluded [10, 24]. 

### Sample size calculation

Sample size was calculated using MedCalc Statistical Software version 19.0.5 (MedCalc Software bvba, Ostend, Belgium; https://www.medcalc.org; 2019). Sample size was calculated assuming 80% study power and 5% alpha error. Aman et al. [21] reported mean (SD) change in root length (mm) after using clear aligners= -0.49 (0.57) and − 0.53 (0.59), in maxillary lateral and central incisors, respectively (mean (SD) % change= -3.72 (4.08) and − 4.16 (4.05), respectively). Based on the comparison of means, using paired *t*-test, the required sample size was calculated to be 23 CBCT scans. This has been increased to 25 to make up for procedural problems [25]. Fifty CBCT scans of 25 adult patients (representing T1 [before treatment] and T2 [after treatment]) (11 males, 14 females; mean age 32.67 ± 10.67 years), who had anterior open bite ranging from 1 to 4 mm and treated with clear aligners, were retrospectively analyzed. Skeletal classification included 21 Class I, 2 Class II, and 2 Class III cases. Dental malocclusions were the same as the skeletal patterns. The class II cases were class II division 1 with no subdivisions in either class II or class III cases.

### Procedures

#### CBCT acquisition and volumetric analysis

**Fig. 1:**
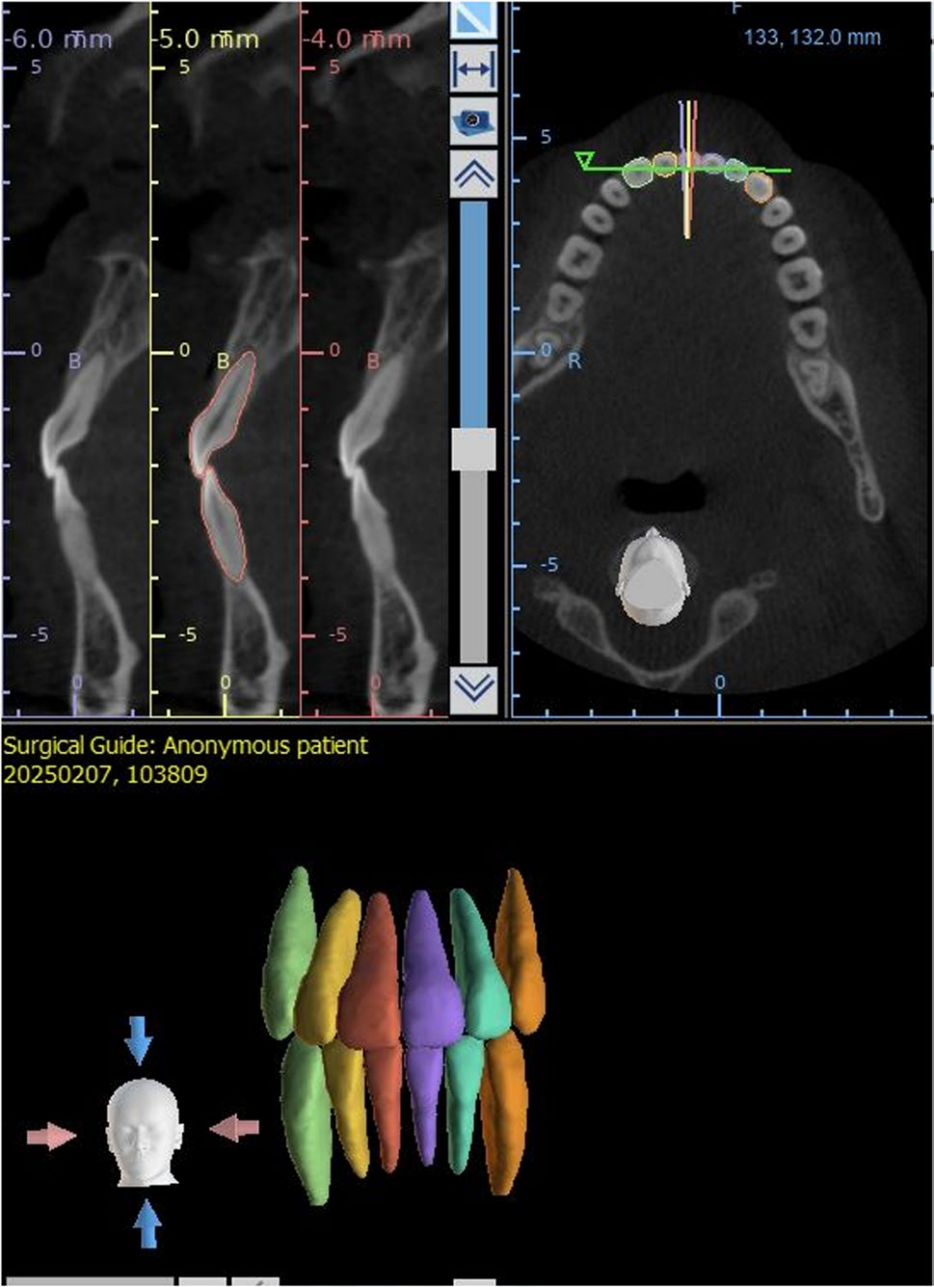
Radiographic evaluation of automatic teeth segmentation performed by Bluesky bio software

Pre-treatment (T1) and post-treatment (T2) CBCT scans were acquired using identical parameters (120 kVp, 10 mA, 13 × 16 cm FoV, 0.3 mm voxel size, 8.9 s exposure time) using the i-CAT Next Generation system (Imaging Sciences International, Hatfield, PA). The DICOM datasets were processed using Blue Sky Plan 4 (Blue Sky Bio LLC, Libertyville, IL) for semi-automated segmentation **(**Fig. 1**).**

**Fig. 2:**
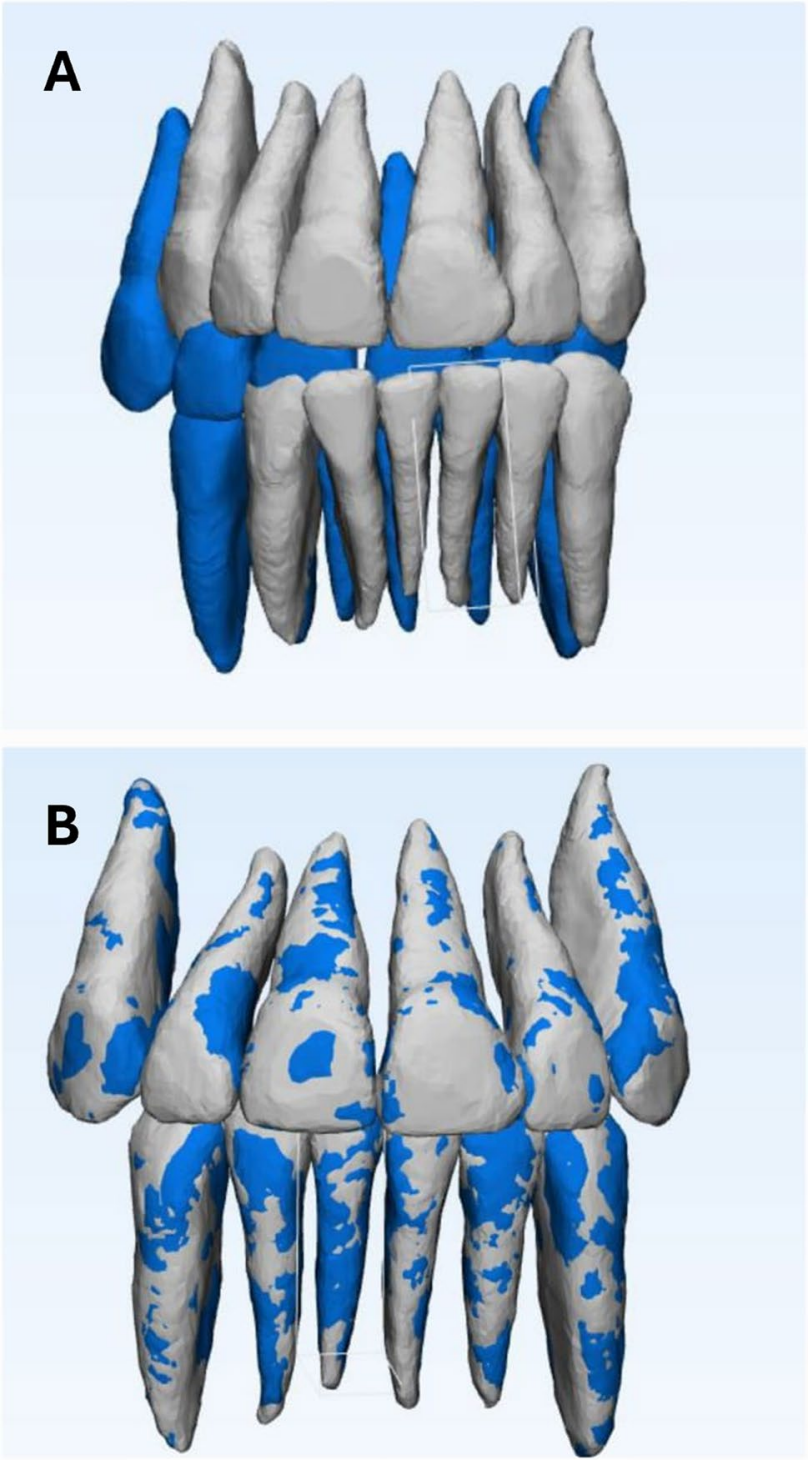
Superimposition of tooth positions before and after treatment. Gray models represent the pre-treatment tooth positions, whereas blue models indicate the post-treatment tooth positions. **A** Pre-superimposition. **B** Post-superimposition

For each CBCT, twelve (upper and lower anterior teeth [canine to canine]) were segmented at T1 and T2, exported as Standard Tessellation Language (STL) files, and superimposed using 3-matic software (3-matic Research 13.0, Materialise NV, Leuven, Belgium) **(**Fig. 2**).**

Automated segmentation was initially used to isolate the dental structures from the surrounding tissues. Due to the potential similarity in density between dental roots and adjacent cortical bone, manual refinement was performed when required using the software’s editing tools to ensure accurate separation of the tooth roots from the alveolar bone. Initial superimposition between the datasets was performed in 3-matic software using a three-point (N-point) point-based registration protocol [26]. Three anatomical landmarks on the tooth crown were selected to achieve the initial alignment: one point on the mesial surface, one on the distal surface, and a third point located at the level of the cemento-enamel junction. Following the initial point-based alignment, global registration was applied to further refine the superimposition between the datasets. Because global registration using the entire tooth may potentially mask subtle root changes, the alignment was carefully evaluated with particular attention to crown alignment, which represents the most stable structure for superimposition in longitudinal root assessment. When minor discrepancies were detected, manual refinement of the superimposition was performed to ensure optimal crown-based alignment prior to the final measurements.

**Fig. 3:**
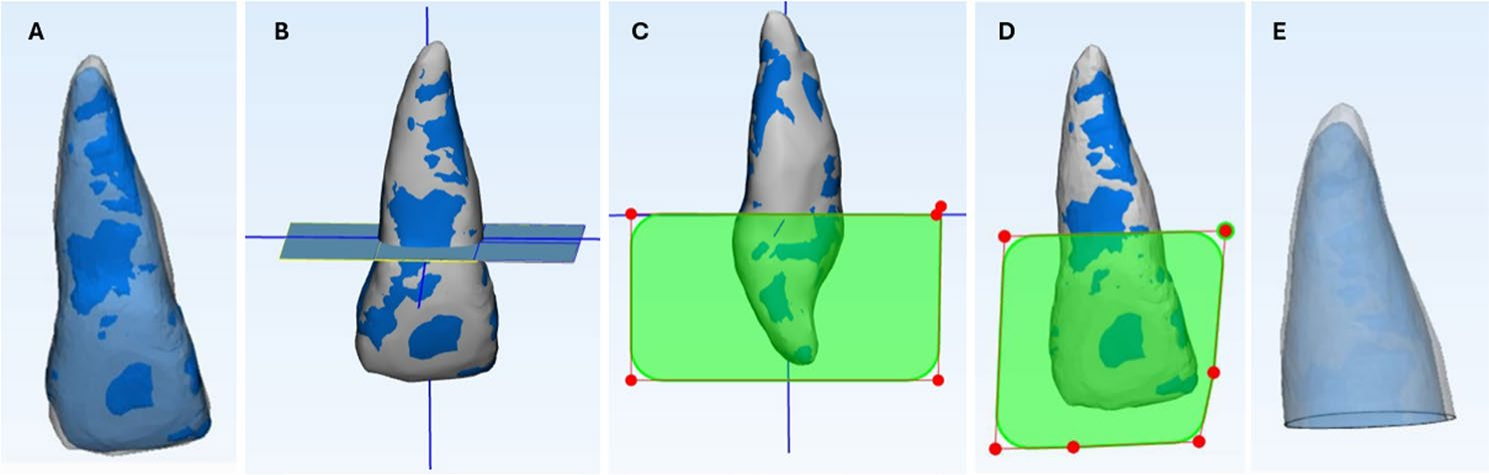
Workflow for volumetric root assessment. **A** Superimposition of the tooth before and after treatment (gray: T1; blue: T2). **B** Three-dimensional view of crown sectioning at the reference plane. **C** Crown sectioning at the reference plane in the proximal view. **D** Crown sectioning at the reference plane in the labial view. **E** Superimposed root volume after crown removal

To standardize crown–root separation, a trim plane was created at the level of the cementoenamel junction (CEJ). This plane was oriented perpendicular to the long axis of the tooth to ensure consistent trimming across all the samples. The long axis of the tooth was defined by connecting the central point of the crown to the apical region of the root, representing the overall anatomical axis of the tooth. The trim plane was then positioned to intersect the tooth at the CEJ level while maintaining a perpendicular orientation to the defined long axis **(**Fig. 3**).** Only the root portion was then computed to assess the change in root volume between the two time points using the following equation: % change = [(T2 − T1) / T1] × 100.

#### Cephalometric analysis

**Fig. 4:**
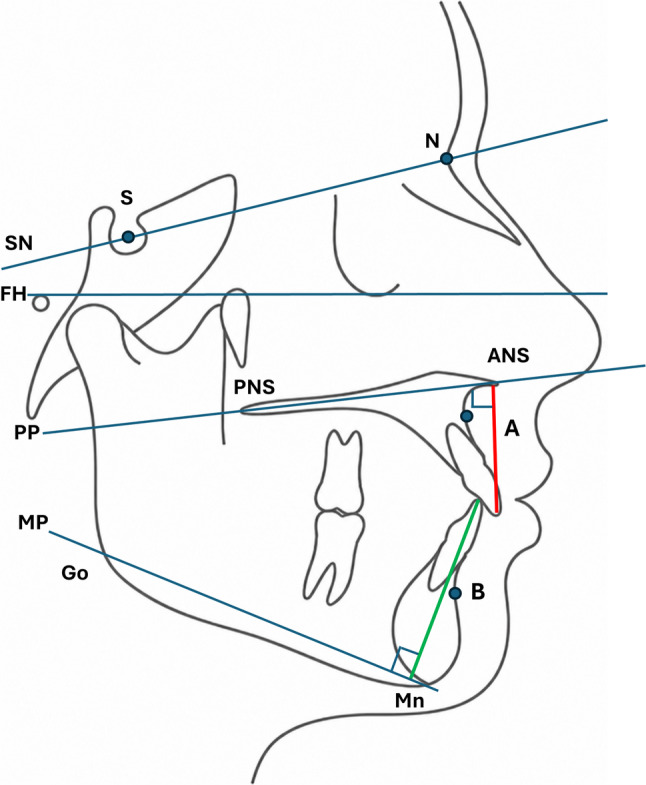
Landmarks and reference planes used for cephalometric analysis. SN: Sella–Nasion plane; FH: Frankfort horizontal plane; PP: Palatal plane; MP: Mandibular plane. Red line: U1–PP (Linear distance between Upper central incisor tip and palatal plane). Green line: L1–MP (Linear distance between lower central incisor tip and mandibular plane)

Lateral cephalometric radiographs were generated from (T1) and (T2) CBCT scans using Dolphin 3D Imaging software (Dolphin Imaging and Management Solutions, Chatsworth, CA, USA) to obtain skeletal and dental measurements before and after treatment. The measured parameters are defined in Table 1, and the landmarks and reference planes are illustrated in Fig. 4.

**Table 1:** Skeletal and dental measurements

SNA (°)	Angle between Sella (S), Nasion (*N*), and A Point, indicating the anteroposterior position of the maxilla relative to the anterior cranial base.
SNB (°)	Angle between Sella (S), Nasion (N), and B Point, indicating the anteroposterior position of the mandible relative to the anterior cranial base.
ANB (°)	Angle between A Point, Nasion (N), and B Point, indicating the skeletal relationship between the maxilla and mandible.
FH-MP (°)	Angle between Frankfort horizontal plane and mandibular plane.
U1-FH (°)	Internal angle between long axis of upper central incisor and Frankfort horizontal plane.
L1-MP (°)	Internal angle between long axis of lower central incisor and mandibular plane.
U1-PP (mm)	Linear distance between Upper central incisor tip and palatal plane.
L1-MP (mm)	Linear distance between lower central incisor tip and mandibular plane.

The linear measurements U1–PP (mm) and L1–MP (mm) were used to assess the vertical positional changes (extrusion) of the maxillary and mandibular incisors, respectively, during treatment, allowing the evaluation of their potential relationship with volumetric root changes.

SN: Sella–Nasion plane; FH: Frankfort horizontal plane; PP: Palatal plane; MP: Mandibular plane. Red line: U1–PP (Linear distance between Upper central incisor tip and palatal plane to the palatal plane). Green line: L1–MP (Linear distance between lower central incisor tip and mandibular plane).

#### Masking

The timing of record acquisition, before or after treatment, can be determined using CBCT. Consequently, the orthodontist who assessed the results could not be blinded. However, the statistician remained blinded throughout the data analysis process.

#### Reliability

Although final volumetric measurements from CBCT scans were computed using software algorithms, the preceding workflow involved several operator-dependent steps, including segmentation refinement, image registration, landmark selection, and crown–root separation. To assess the reproducibility of this workflow, 10 randomly selected CBCT datasets were re-evaluated by two examiners (D.E. and E.A.) after a two-week interval. In addition, cephalometric measurements were performed independently by the same examiners on 10 randomly selected lateral cephalograms to evaluate intra- and inter-examiner reliability using the intraclass correlation coefficient (ICC) [27]. 

#### Statistical analysis

Normality was tested using descriptive statistics, Q-Q plots, histograms, and Shapiro Wilk normality test. Descriptive data are presented as means and standard deviations (SD). For each tooth type, the values were calculated as the average of the corresponding bilateral teeth (left and right). Root volume changes pre- and post-treatment were compared using a paired t-test with calculation of mean differences and 95% confidence intervals (CIs). Percent change in root volume was calculated using the following formula:

% Change = [(T2 − T1) / T1] × 100. Gender differences in root resorption were evaluated using Mann-Whitney U test, and correlation with age was assessed using Pearson’s correlation. Also, correlations between key cephalometric parameters and changes in root volume were assessed using Pearson’s correlation coefficient. Comparisons among centrals, laterals, and canines were performed using repeated measures ANOVA or Friedman test according to the variable normality, followed by multiple pairwise comparisons using Bonferroni corrected significance level. Significance was set at p-value < 0.05. Data were analyzed using IBM SPSS for Windows (Version 26.0).

## Results

Excellent intra- and inter-examiner reliability was observed for both CBCT-derived volumetric measurements and lateral cephalometric analyses (ICC = 0.90–0.94 and 0.92–0.96, respectively; *p* < 0.001), indicating high reproducibility of the measurement procedures [27]. 

The sample comprised 25 adults (11 males, 14 females; mean age 32.67 ± 10.67 years) with anterior open bite (1–4 mm), including 21 Class I, 2 Class II, and 2 Class III cases. Baseline cephalometric measurements were as follows: ANB, 1.30°± 2.83°; U1–FH, 117.33°± 6.88°; and L1–MP, 94.70°± 10. 38°. In addition, the mean treatment duration was 25.46 ± 9.58 months.

Table 2 shows descriptive statistics for average root volume pre-(T1) and post-(T2) treatment. Volumetric root reduction was statistically significant in the maxillary central incisors (− 10.87 ± 10.10), lateral incisors (− 7.98 ± 5.10), and canines (− 15.77 ± 7.68), with a significant difference among the groups (*p* < 0.001). Similarly, significant reductions were observed in the mandibular central incisors (− 5.31 ± 2.62), lateral incisors (− 7.69 ± 4.39), and canines (− 15.22 ± 4.57), also demonstrating intergroup significance (*p* < 0.001). The canines exhibited the greatest volumetric reduction in both arches (*p* < 0.001).

**Table 2:** Comparison of average root volume (mm^3^) pre- and post-treatment

Arch	Tooth	Pre-treatment	Post-treatment	Difference (Post–Pre)	95% CI	Percent change Mean (SD) (%)	Within-group p-value†
		Mean (SD) (mm³)					
Upper	11 and 21	188.12 (39.28)ᵃ	177.25 (39.77)ᵃ	-10.87 (10.10)ᵃ	-13.74, -8.00	-5.91 (4.93)ᵃ	< 0.001*
	12 and 22	149.15 (42.42)ᵇ	141.17 (41.22)ᵇ	-7.98 (5.10)ᵇ	-9.43, -6.53	-5.50 (3.24)ᵇ	< 0.001*
	13 and 23	260.82 (82.57)ᶜ	245.05 (81.13)ᶜ	-15.77 (7.68)ᶜ	-17.94, − 13.58	-6.33 (2.58)ᶜ	< 0.001*
	**Between-group p-value‡**	< 0.001*	< 0.001*	< 0.001*	—	< 0.001*	—
Lower	31 and 41	112.70 (26.06)ᵃ	107.40 (25.58)ᵃ	-5.31 (2.62)ᵃ	-6.05, -4.56	-4.86 (2.54)ᵃ	< 0.001*
	32 and 42	141.55 (31.50)ᵇ	133.86 (31.17)ᵇ	-7.69 (4.39)ᵇ	-8.94, -6.44	-5.58 (3.20)ᵇ	< 0.001*
	33 and 43	235.84 (67.38)ᶜ	220.62 (65.98)ᶜ	-15.22 (4.57)ᶜ	-16.52, -13.92	-6.75 (2.33)ᶜ	< 0.001*
	**Between-group p-value‡**	< 0.001*	< 0.001*	< 0.001*	—	< 0.001*	—

*SD* Standard Deviation, *CI* Confidence interval

† Within-group p-value: Paired t-test comparing pre- and post-treatment values

‡ Between-group p-value: Repeated-measures ANOVA was used for comparisons between central incisors, lateral incisors, and canines in pre- and post-treatment values, while comparisons between these teeth in the difference and percent change were performed using the Friedman test

*statistically significant at p-value < 0.05

a-c: different letters denote significant differences between centrals, laterals, and canines using Bonferroni correction

All teeth demonstrated a reduction in the root volume percentage, as presented in Table 2. In the maxillary arch, canines showed the greatest percent change (− 6.33 ± 2.58), followed by central incisors (− 5.91 ± 4.93) and lateral incisors (− 5.50 ± 3.24). Similarly, in the mandibular arch, canines exhibited the highest percent change (− 6.75 ± 2.33), followed by lateral incisors (− 5.58 ± 3.20) and central incisors (− 4.86 ± 2.54).

Male patients exhibited significantly greater root volume reduction than female patients across all anterior teeth (Mann–Whitney U test, *p* < 0.05; Table 3). No significant correlation was observed between age and the percentage of root volume change (*p* > 0.05; Table 4). Table 5 presents the changes in skeletal cephalometric measurements. No significant differences were observed in SNA, SNB, or ANB (− 0.37°± 0.88°, − 1.17°± 4.79°, and − 0.16°± 0.40°, respectively; *p* > 0.05). In contrast, a significant reduction was observed in the FH–MP angle (− 0.95°± 0.75°; *p* < 0.001).

**Table 3:** Comparison of percent change in root volume between males and females

Arch	Tooth	Male	Female	P value
		Mean (SD)		
Upper	11 and 21	-5.28 (2.06)	-2.76 (1.77)	< 0.001*
	12 and 22	-7.48 (3.81)	-4.49 (2.94)	0.005*
	13 and 23	-7.69 (2.60)	-5.60 (2.18)	0.004*
Lower	31 and 41	-6.10 (2.68)	-3.88 (1.97)	< 0.001*
	32 and 42	-7.67 (2.10)	-3.93 (2.97)	< 0.001*
	33 and 43	-8.14 (2.45)	-5.69 (1.58)	< 0.001*

Mann-Whitney U test was used

*SD* Standard Deviation

*statistically significant at p-value < 0.05

**Table 4:** Correlation of age and percent change in root volume

Arch	Tooth	*r* value	*p* value
Upper	11 and 21	-0.21	0.14
	12 and 22	-0.11	0.46
	13 and 23	-0.11	0.45
Lower	31 and 41	0.18	0.22
	32 and 42	0.15	0.29
	33 and 43	0.06	0.69

*r* correlation coefficient

*statistically significant at p-value < 0.05

**Table 5:** Comparison of skeletal readings pre- and post-treatment

Parameter	Pre-treatment (T1)	Post-treatment (T2)	Difference (T2-T1)	95% CI	*P* value
	Mean (SD)				
SNA (°)	78.82 (4.79)	78.45 (4.46)	-0.37 (0.88)	-0.75, 0.01	0.10
SNB (°)	77.52 (4.22)	76.35 (6.18)	-1.17 (4.79)	-3.24, 0.90	0.25
ANB (°)	1.30 (2.83)	1.14 (2.73)	-0.16 (0.40)	-0.33, 0.02	0.10
FMA (°)	28.80 (4.39)	27.86 (4.18)	-0.95 (0.75)	-1.27, -0.62	< 0.001*

*SD* Standard Deviation, *CI* Confidence interval

Paired t-test was used

*statistically significant at p-value < 0.05

Table 6 presents the changes in dental cephalometric measurements. Significant reductions were observed in the U1–FH and L1–MP angles (− 4.20 ± 4.70° and − 4.82 ± 4.43°, respectively; *p* < 0.001). A statistically significant increase was observed in the linear measurements of U1–PP (mm) and L1–MP (mm) (0.64 ± 0.16 mm and 0.84 ± 0.13 mm, respectively; *p* < 0.001).

**Table 6:** Comparison of dental readings pre- and post-treatment

Parameter	Pre-treatment (T1)	Post-treatment (T2)	Difference (T2-T1)	95% CI	*P* value
	Mean (SD)				
U1-FH (°)	117.33 (6.88)	113.13 (4.51)	-4.20 (4.70)	-6.23, -2.17	< 0.001*
L1-MP(°)	94.70 (10.38)	89.88 (8.03)	-4.82 (4.43)	-6.74, -2.91	< 0.001*
U1-PP (mm)	30.14 (1.75)	30.78 (1.74)	0.64 (0.16)	0.57, 0.71	< 0.001*
L1-MP (mm)	38.00 (1.69)	38.84 (1.64)	0.84 (0.13)	0.78, 0.89	< 0.001*

*SD* Standard Deviation, *CI* Confidence interval

Paired t-test was used

*statistically significant at p-value < 0.05

No significant correlation was found between U1–PP (mm) and the change in root volume of U1, nor between U1–FH angle and the root volume change of L1 (Table 7).Similarly, no significant correlation was observed between L1–MP (mm) and the change in root volume of U1, nor between L1–MP angle and the root volume change of L1 (Table 7).

**Table 7:** Correlation between root volume change and key cephalometric parameters

Tooth	Cephalometric parameter	*r* value	*P* value
11 and 21	U1-PP (mm)	-0.12	0.60
	U1-FH **(°)**	0.15	0.52
31 and 41	L1-MP (mm)	0.18	0.43
	L1-MP (°)	-0.10	0.70

*r* correlation coefficient

## Discussion

Patients with open bite have shown successful treatment using clear aligners [28]. Due to limited data on external root resorption in such cases [29], this study compared anterior teeth root volume changes using CBCT in open bite patients treated with clear aligners. Based on the study findings, the null hypothesis was rejected, as statistically significant root volume reduction was observed in all anterior teeth.

The use of CBCT with semiautomated segmentation technique provides the advantage of assessing changes in root morphology volumetrically, which previously proved to provide better knowledge of root alterations during treatment [16, 30]. 

The volumetric root reduction observed in this study (around 5–6% across anterior teeth) is consistent with findings from recent clear aligner research, including the study by Rossi et al. [16], which reported comparable volumetric changes. Previous CBCT-based studies—primarily based on linear measurements—have described typical OIERR in the range of 3–8% [31, 32]. A mean volumetric reduction of 5–6% is considered mild and clinically insignificant, as the literature indicates that root volume losses below approximately 10% are unlikely to significantly affect tooth stability or periodontal support [20]. 

Our study showed a significant decrease in average root volume for maxillary and mandibular anterior teeth. In the maxillary arch, canines exhibited the greatest reduction, followed by central incisors and lateral incisors, consistent with the calculated percentage changes. This pattern may be explained by the specific biomechanics of clear aligner therapy (CAT) in open bite correction. Vertical elastics applied between the maxillary and mandibular canines, together with attachments and staged aligner activation, may increase apical stress concentration and cyclic loading within the periodontal ligament, thereby contributing to root resorption. Furthermore, aligners deliver programmed, staged tooth movements using continuous light forces with repeated activation cycles, which may result in intermittent compressive stress within the periodontal ligament. These cyclic loading patterns can stimulate clastic cell activity, thereby contributing to root resorption [2, 33]. 

Similarly, Rossi et al. [16] reported that maxillary central incisors showed a higher decrease in average root volume than the lateral incisors in a study treating malocclusion using CAT. This arch-specific variation may be attributed to differences in root morphology [34], occlusal loading, and functional adaptations, including tongue posture in open bite patients [35]. In particular, teeth with unfavorable root morphologies—such as long, narrow, or dilacerated roots—have been shown to be more susceptible to external root resorption due to increased stress concentration along the root surface and reduced resistance to orthodontic forces, as reported by Mirabella and Årtun [34]. 

In contrast, Sameshima and Sinclair [5] reported that, during fixed orthodontic treatment, maxillary canines experienced greater root volume reduction than incisors, while maxillary lateral incisors exhibited greater reduction than central incisors. These differences could be due to the differences in force delivery and biomechanics may influence the magnitude and distribution of stresses along the root surface, thereby contributing to variations in root resorption patterns. Furthermore, differences in malocclusion characteristics, along with the higher prevalence of atypical root morphologies in maxillary lateral incisors—such as pipette-shaped, pointed, or dilacerated roots—may further contribute to the observed variations in root resorption patterns [34]. 

The mandibular arch exhibited a different pattern of root volume change. Although canines showed the highest reduction, lateral incisors had a greater root volume loss than central incisors. This pattern, supported by percentage changes, is likely multifactorial. In open bite patients, altered tongue posture or anterior tongue thrust [35, 36] may modify occlusal loading and disrupt force equilibrium, leading to increased or prolonged stress on the mandibular anterior teeth during orthodontic treatment, which may contribute to root resorption. In addition, variations in root morphology [34], alveolar bone characteristics, initial tooth inclination, and the magnitude and direction of orthodontic forces [37, 38], may further influence stress distribution along the root surface, thereby affecting susceptibility to OIERR. These findings highlight the multifactorial nature of orthodontically OIERR, where both biomechanical and functional factors interact to influence treatment outcomes.

Maxillary and mandibular canines exhibited the greatest reduction in the average root volume. This may be related to the use of vertical elastics between the upper and lower canines as a part of open bite correction, similar to approaches such as the multiloop edgewise archwire (MEAW) technique [39]. This results in complex force systems and altered force vectors across the dentition. Although extrusion is generally associated with a lower risk of root resorption, in this context it is combined with continuous elastic wear, attachments, and staged aligner activation, resulting in more complex force systems. These combined mechanics may have increased stress concentration at the apical region and generate cyclic loading of the periodontal ligament, thereby contributing to the observed pattern of root resorption.

This study demonstrated a statistically significant percent decrease in root volume in males than in females. This agrees with Aman et al. [21], where sex was considered as a risk factor for OIERR, with males experienced higher external root resorption than females. A possible explanation previously suggested is higher bone density in males, which may be associated with prolonged treatment duration and increased resorptive response [40]. However, other studies reported no significant difference between males and females regarding OIERR [41, 42]. A recent cross-sectional study addressing gender-specific treatment effects in orthodontic treatment concluded that gender did not have a specific effect on orthodontic outcomes in the included studies [43]. The majority of outcomes showed non-significant gender effects overall for at least 50% of the studies examined. Accordingly, the present findings should be interpreted cautiously in the context of conflicting evidence and may reflect sample-specific variability rather than a definitive biological sex-related effect.

No significant correlation was noted between age and root volume changes. This agrees with Pastro et al. [44] who postulated that initial age does not significantly affect root resorption.

There was a significant decrease in the FH-MP, which could be explained by the mechanics used for intruding upper and lower molars, which controlled the vertical dimension and increased the overbite. Harris et al. [45] reported that anterior open bite closure with clear aligners results from a combination of maxillary and mandibular incisors extrusion, molar intrusion, and slight mandibular autorotation, consistent with the mechanics in the present study. Interestingly, one study compared the treatment of open bite cases with clear aligners to a group with fixed appliances in hyperdivergent patients, including those treated with extractions and TADs in the fixed appliance group. The two groups had similar treatment outcomes, indicating that CAT may be as successful as fixed appliances with auxiliaries in vertical dimension management [46]. 

Moreover, U1–FH and L1–MP angles decreased significantly, indicating retroclination of both maxillary and mandibular incisors. In the included open bite cases, treatment was primarily directed toward posterior tooth intrusion and counterclockwise mandibular rotation. This often resulted in anterior interferences or a tendency toward anterior crossbite, necessitating planned lingual movement of the lower incisors. Such movement was achieved through lower arch interproximal reduction (IPR) in the anterior segment. Furthermore, the use of attachments and controlled staging may enhance torque control and facilitate root movement. Similar findings have been reported by Harris et al. [45], who demonstrated significant retroclination of both maxillary and mandibular incisors in anterior open bite patients treated with clear aligners.

Additionally, the linear measurements U1–PP and L1–MP increased significantly, indicating that both maxillary and mandibular incisors underwent significant extrusion during treatment. This agrees with previous findings by Harris et al. [45], who also reported significant extrusion of both maxillary and mandibular incisors in the same treated malocclusion using clear aligners.

No significant correlations were identified between the extent of incisors extrusion and root volume changes. Specifically, neither U1–PP (mm) nor L1–MP (mm) showed a significant correlation with the degree of root resorption. Similarly, angular changes reflecting the inclination of the incisors, including U1–FH and L1–MP angles, were not significantly correlated with root volume changes. These findings suggest that, within the limitations of the present study, the magnitude of incisor extrusion and inclination may not be the primary determinants of root resorption in anterior open bite patients treated with clear aligners. This observation is consistent with previous reports indicating that root resorption is a multifactorial phenomenon, influenced not only by the amount of tooth movement but also by individual biological variability, genetic predisposition, and treatment-related factors such as force magnitude [47, 48]. 

Visual assessment suggested that root reduction was more pronounced in the apical region than along the external root surfaces. However, as this study focused on overall volumetric changes rather than region-specific analysis, further studies are warranted to quantitatively evaluate the spatial distribution of root resorption, particularly comparing apical and lateral root surfaces.

### Strengths and limitations

The primary strength of this study is the use of CBCT-based volumetric assessment, which provides superior sensitivity compared with two-dimensional linear measurements. Standardized treatment protocols and consistent imaging parameters further strengthen internal validity.

Limitations include the retrospective design and the absence of a fixed appliance group for comparison, which restricts the ability to determine relative differences in external root resorption between treatment modalities. The sample also demonstrated heterogeneity in patient age, which may have introduced variability in treatment response. In addition, the sample size was based on linear root length changes rather than volumetric measurements due to limited available data, which may affect the precision of the estimation. Furthermore, the relatively small sample size and lack of multivariable analysis to control for confounders such as treatment duration, tooth movement magnitude, and root morphology may have influenced the findings. Accordingly, the observed sex-related differences should be interpreted with caution and considered exploratory rather than conclusive.

## Conclusions

Open bite patients treated with clear aligners showed mild volumetric root reduction in all anterior teeth, generally considered clinically insignificant based on available literature. Patterns of external root resorption varied by arch and tooth type, with canines showing the greatest reduction, followed by maxillary central incisors and mandibular lateral incisors within their respective arches. Male patients exhibited significantly greater root volume loss, whereas age was not significantly correlated with root reduction. Clear aligner therapy also achieved effective vertical control, with significant retroclination and extrusion of both maxillary and mandibular incisors. No significant correlations were identified between the extent of incisor extrusion or inclination and root volume changes. Accordingly, the null hypothesis was rejected.

## Data Availability

The datasets used and/or analyzed during the current study are available from the corresponding author upon reasonable request.

## Abbreviations

OIERR: Orthodontically induced external root resorption
CAT: Clear aligners therapy
IPR: Interproximal reduction
CBCT: Cone-Beam Computed Tomography
T1/T2: Pre- and Post-Treatment Time Points
FH-MP: Frankfort-Mandibular Plane Angle
CEJ: Cementoenamel Junction
STL: Standard Tessellation Language
